# Bemessungsempfehlungen zur Invalidität in der privaten Unfallversicherung – fachübergreifender Konsens – Stand 09/2024: Von der Fachgesellschaft Interdisziplinäre Medizinische Begutachtung (FGIMB) e. V. zur Anwendung empfohlen am 06.10.2023

**DOI:** 10.1007/s00113-024-01483-5

**Published:** 2024-09-18

**Authors:** H. T. Klemm, E. Ludolph, W. Willauschus, M. Wich, S. Weber, R. Fuhrmann, T. Heintel

**Affiliations:** 1 Fachgesellschaft Interdisziplinäre Medizinische Begutachtung e. V., Hamburg, Deutschland; 2 Freies Institut für medizinische Begutachtungen Bayreuth/Erlangen (FIMB), Ludwigstraße 25, 95444 Bayreuth, Deutschland; 3 Institut für ärztliche Begutachtung, Düsseldorf, Deutschland; 4 Gutachteninstitut Orthopädisch-unfallchirurgische Praxisklinik alphaMED, Bamberg, Deutschland; 5 Klinik für Unfallchirurgie und Orthopädie, BG-Klinikum Unfallkrankenhaus Berlin, Berlin, Deutschland; 6 Abteilung für Unfallchirurgie und Orthopädie, Sana Klinikum Dahme-Spreewald GmbH Achenbach Krankenhaus, Königs Wusterhausen, Deutschland; 7 Klinik für Orthopädie, Unfallchirurgie und Rehabilitative Medizin, Universitätsmedizin Greifswald, Greifswald, Deutschland; 8 Wissenschaftlicher Beirat der FGIMB, Deutsche Gesellschaft für Handchirurgie, Berlin, Deutschland; 9 Wissenschaftlicher Beirat der FGIMB, Deutsche Assoziation Fuß und Sprunggelenk, Berlin, Deutschland; 10 Klinik für Fuß- und Sprunggelenkchirurgie, Rhön-Klinikum Campus Bad Neustadt, Bad Neustadt, Deutschland; 11 Klinik und Poliklinik für Unfall‑, Hand‑, Plastische und Wiederherstellungschirurgie, Universitätsklinikum Würzburg, Würzburg, Deutschland; 12 Wissenschaftlicher Beirat der FGIMB, Deutsche Wirbelsäulengesellschaft, Ulm, Deutschland

**Keywords:** Unfallversicherung, Gliedertaxe, Konsentierte Bemessungsempfehlungen, Invaliditätsbemessung, Beeinträchtigung körperlicher Leistungsfähigkeit, FGIMB, Accident insurance, Disability assessment, Consensus benchmark recommendations, Limb tarifs, Impairment of physical performance, FGIMB

## Abstract

Grundlage der Leistung eines privaten Unfallversicherers ist die Invaliditätsleistung, die ärztlich fristgerecht festzustellen ist. Der Versicherer gibt pauschalierte Sätze der Gliedertaxe für Verlust oder Funktionsunfähigkeit vor, und der ärztliche Sachverständige muss dann auf allgemein anerkannte Bemessungsempfehlungen zurückgreifen können, um den vorgegebenen Rahmen auf die konkrete, individuelle Situation des Versicherten anwenden zu können. Es handelt sich in dieser Arbeit um fachübergreifend konsentierte Eckwerte der Invaliditätsbemessung, die Grundlage einer einheitlichen ärztlichen Begutachtung von unfallbedingten Funktionsbeeinträchtigungen in der privaten Unfallversicherung sein sollen.

Mit Veröffentlichung dieser Bemessungsempfehlungen der FGIMB werden die Empfehlungen von Schröter und Ludolph aus 2009 [12] zurückgenommen, sodass jetzt die Bemessungsempfehlungen der FGIMB an deren Stelle maßgeblich sind.

## Abstract (en)

The centerpiece in private accident insurance is the compensation in cases of disability, which must be determined by a physician within a certain time limit. The insurer contract specifies the compensation rate for loss or inability to function. In cases of insurance the medical expert has to refer to generally accepted revised or updated assessment recommendations in order to be able to apply the given framework to the specific individual disability situation of the insured person. This article deals with the interdisciplinary consensus benchmarks for the assessment of disability, which form the principles of a uniform medical assessment of accident-related functional disorders in the private accident insurance.

With the publication of these new assessment recommendations developed under the guidance of the Specialist Society of Interdisciplinary Medical Expert Opinion (FGIMB), the recommendations published by Schröter and Ludolph in 2009 [12] are withdrawn, so that these are now replaced as the authoritative version by the assessment recommendations of the FGIMB.

## Vorbemerkungen

Die Bemessungsempfehlungen (Publikation zu den Grundlagen erhältlich bei Springer 10.1007/s00113-023-01344-7) wurden erarbeitet unter Beteiligung ärztlicher Fachgesellschaften, mit gutachtlicher Materie vertrauter Institutionen und Personen aus Deutschland, Österreich und der Schweiz. Eine ausführliche Darstellung über die Erarbeitung und die konkreten Begründungen zu den Einzelwerten erfolgte bereits in einer Grundlagenpublikation [4–7].

Die vorliegenden Bemessungsempfehlungen werden in Abständen evaluiert und aktualisiert. Eine Arbeitsgruppe analysiert Hinweise von Anwendern zu evtl. Wertungswidersprüchen und nimmt ggf. Korrekturen oder Ergänzungen vor. Während in dieser Arbeit die Eckwerte vorzugsweise in Tabellenform dargestellt sind, ist die jeweils aktuelle Version online visualisiert und erreichbar unter www.invaliditaet-online.de [3]; dort werden alle Gelenkstellungen von unserem 3D-Modell (genannt: INVATAR) vorgeführt, sodass Bewertungen auch für Nichtmediziner wie Juristen, Sachbearbeiter und/oder Betroffene nachvollziehbar werden.

Bezugspunkt für die Invaliditätsbemessung sind die *Allgemeinen Unfallversicherungsbedingungen* (Musterbedingungen, herausgegeben vom Gesamtverband der Versicherer GDV [2]).

Die Eckwerte der Invalidität sind zunächst in Tabellenform dargestellt und für den Bereich der Gliedertaxe aufgeteilt in die Komplexe Verlust (A) – Versteifung (B) – Funktionsbeeinträchtigung (C), jeweils beginnend mit dem größten Funktionsverlust. Ebenso sind Eckwerte für Funktionsbeeinträchtigungen außerhalb der Gliedertaxe angegeben.

## Relevanz konsentierter Invaliditätseckwerte

Bei der Begutachtung von Unfallverletzungsfolgen sollen gleiche Funktionsdefizite auch mit gleichen (vergleichbaren) Invaliditätswerten belegt werden. Für die Umsetzung dieses Ziels bedarf es Eckwerten, die nicht eminenzbasiert vorgetragen, sondern mit einem breiten Konsens erarbeitet wurden. Unterhalb der Werte für Gliedmaßenverluste (die durch die AUB vertraglich vorgegeben sind) hat die langjährige Begutachtungspraxis gezeigt, dass eine Umsetzung der tatsächlichen Funktions- und Leistungsbeeinträchtigungen der jeweiligen versicherten Person in eine dauernde Invalidität nur dann vergleichbar, transparent und nachvollziehbar formuliert werden kann, wenn sich diese auf spezifizierte und breit akzeptierte Bemessungsempfehlungen stützt.

Mit Veröffentlichung dieser Bemessungsempfehlungen der FGIMB werden die Empfehlungen von Schröter/Ludolph aus 2009 [12] zurückgenommen, sodass jetzt die Bemessungsempfehlungen der FGIMB an deren Stelle maßgeblich sind.

## Umsetzung der Allgemeinen Unfallversicherungsbedingungen

Bezugspunkt für die Invaliditätsbemessung sind die Allgemeinen Unfallversicherungsbedingungen (Musterbedingungen, herausgegeben vom GDV, Stand 2020 (https://www.gdv.de/resource/blob/6252/f5121ebea18eb5800be7566316330293/01-allgemeine-unfallversicherungsbedingungen-aub-2020-﻿-data.pdf)).

**Abb. 1:**
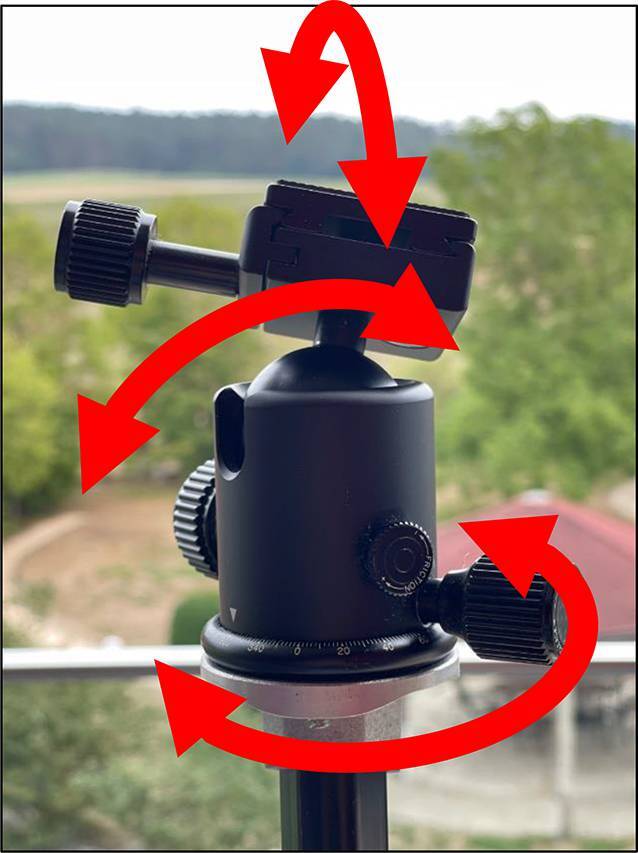
Vergleichende Darstellung einer anatomischen Gelenkbeweglichkeit

Hinzuweisen ist für die Anwendung der Bemessungsempfehlungen, dass es nach den zugrunde liegenden AUB auf eine rein funktionelle Beurteilung der Unfallverletzungsfolgen ankommt. Insbesondere bei der Beurteilung von Funktionsbeeinträchtigungen in Gelenken ist also nicht ausschließlich auf die eingeschränkte Winkelgradzahl der Bewegung im Vergleich zur Norm abzustellen (Abb. 1, 2 und 3).

**Abb. 2:**
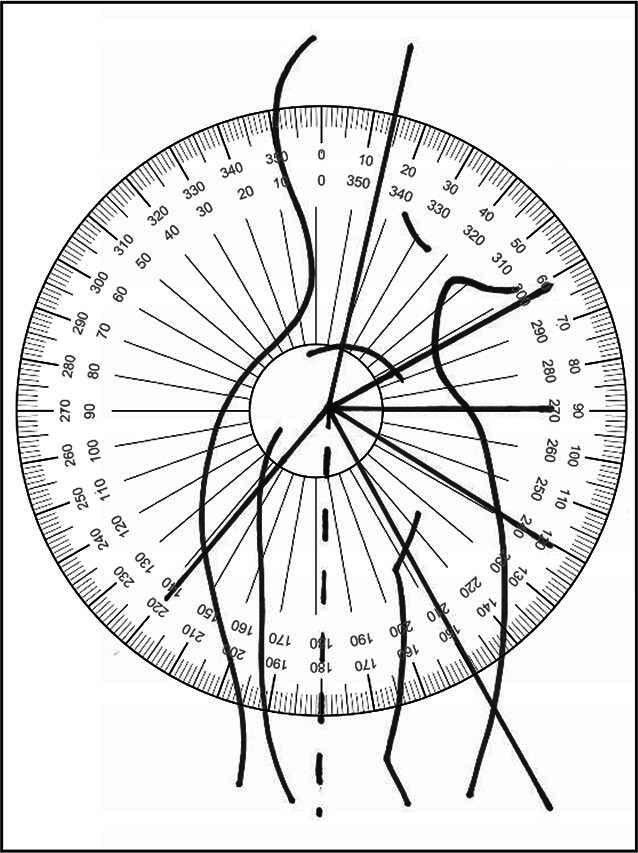
Rein anatomische Betrachtung eines Gelenks mittels Winkelausschlägen

**Abb. 3:**
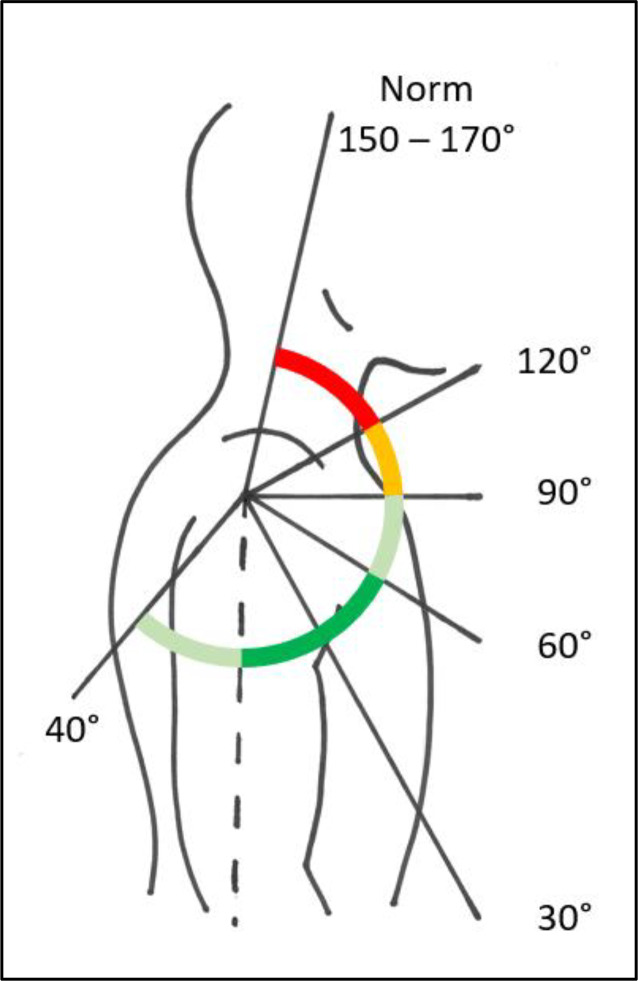
Funktionelle Betrachtung einer Gelenkbeweglichkeit (beispielhaft am Schultergelenk: *dunkelgrün* funktionell von herausragender Bedeutung, *hellgrün* funktionell von großer Bedeutung, *gelb* funktionell von weniger großer Bedeutung, *rot* funktionell von untergeordneter Bedeutung)

Bei dem Kugelgelenk für die Kamera auf einem Fotostativ in Abb. 1 sind eine Drehung von 360° (*unterer Pfeil*), eine Kippung in die Bildebene hinein und heraus von jeweils 45° (*oberer Pfeil*), in der Bildebene nach links von 90° und nach rechts von 45° (*mittlerer Pfeil*) möglich. Für die Fotografie ist sicherlich jede Bewegungsebene in etwa gleichwertig. Unfallbedingte Funktionsbeeinträchtigungen wirken sich jedoch unterschiedlich aus.

Nur das Anlegen eines Winkelmessers an ein Gelenk (Abb. 2) mit Dokumentation der Einschränkung gegenüber der Norm wird der funktionellen Betrachtungsweise nicht gerecht.

In der Physiologie der Gelenkbeweglichkeit, also einer funktionellen Betrachtung, ist zu berücksichtigen, dass der Versicherte – beispielhaft an den oberen Gliedmaßen – erwarten darf, dass die notwendige Bewegung in Blickrichtung nach vorn (Vorwärtshebung des Arms) bis zur Horizontalen eine höhere Wertigkeit erfährt als z. B. die Vorwärtshebung des Arms über 120°. Diese „Wertigkeit“ ist an einer Farbskala von *rot* (funktionell von untergeordneter Bedeutung) bis *grün* (funktionell von herausragender Bedeutung) dargestellt (Abb. 3).

Auch ist insgesamt eine Bewegungseinschränkung in Blickrichtung nach vorn einschneidender als eine Minderung der Abhebefähigkeit des Arms seitlich über die Horizontale.

Die zur Bemessung von Funktionsbeeinträchtigungen angegebenen Werte stellen Eckwerte dar. Bei Unfallbetroffenheit verschiedener Gelenke an einer Gliedmaße ist zunächst das herausragendste Funktionsdefizit zu benennen und anschließend subsumierend zu betrachten, ob aus weiteren Unfallfolgen Änderungen der Invaliditätsbemessung (sowohl erhöhend als auch erniedrigend) resultieren.

Die Eckwerte der Invalidität sind zunächst in Tabellenform dargestellt und für den Bereich der Gliedertaxe aufgeteilt in die Komplexe Verlust (A), Versteifung (B) und Funktionsbeeinträchtigung (C), jeweils also beginnend mit dem größten Funktionsverlust. Ebenso sind Eckwerte für Funktionsbeeinträchtigungen außerhalb der Gliedertaxe angegeben.

Zur Vereinheitlichung der Bezeichnungen sprechen wir im Vergleich zur Originalpublikation zu den Werten der Gelenkversteifung [9] nicht mehr von einer gebrauchs-, sondern funktionsgünstigen Stellung. Im Bereich der Gliedertaxe sind die Invaliditätswerte einheitlich in Zwanzigsteln angegeben.

Zur Nutzung der Bemessungsempfehlungen auch durch Nichtmediziner wird weitestgehend auf lateinische Ausdrücke verzichtet bzw. werden diese in Klammern erklärt.

## Anmerkung zur Vergleichbarkeit von Invaliditätswerten

Bei der Plausibilitätsprüfung von Bemessungseckwerten ist der Kliniker bei Gesamtbetrachtung des Individuums gewillt, Funktionsbeeinträchtigungen z. B. am Daumen mit der Handfunktion oder am Fuß mit der Beinfunktion zu vergleichen. Diese Herangehensweise ist aber zum Scheitern verurteilt, da vom Versicherer die Verlustwerte als Bezugspunkt vorgegeben sind. Betrachtet man sich also den Verlust des Arms im Vergleich zum Beinverlust, so zieht der Armverlust wesentlich mehr Funktionsbeeinträchtigungen nach sich als der Beinverlust, ist aber nach AUB-Musterbedingungen gleichwertig mit 70 % zu bemessen. Insofern kann nur eine Vergleichbarkeit von Werten innerhalb der Gliedmaße/des Gliedmaßenteils erfolgen. Beispielhaft ist also eine Handfunktionsbeeinträchtigung nur mit anderen Funktionsbeeinträchtigungen der Hand vergleichbar und eine Daumenfunktionsbeeinträchtigung nur mit einer anderen am Daumen.

## Normalbeweglichkeit eines Gelenks

Zur Beurteilung von Funktionsbeeinträchtigungen eines Gelenks ist das Wissen über die Normalbeweglichkeit desselben unumgänglich. Dabei sind z. B. beim Handgelenk Globalfunktionen in allen Bewegungsrichtungen zu wichten, während bei anderen Gelenken eine Bewegungsebene eine herausragende Bedeutung hat, wie z. B. im Schulter- oder im Hüftgelenk die Bewegung in Blickrichtung, auf die dann in der Regel die Invalditätsbemessung abstellt, sofern nicht beurteilungsrelevante Beeinträchtigungen in den weiteren Bewegungsebenen vorliegen (Tab. 1).

**Tab. 1:** Die wichtigsten Gelenk-Normbeweglichkeiten

*Schultergelenk* Arm rückwärts/vorwärts 40/0/150–170°	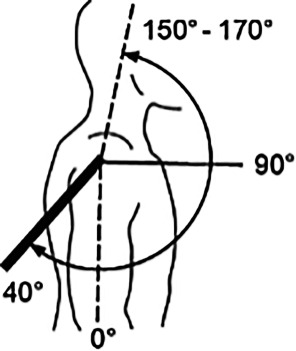
*Ellenbogengelenk* Streckung/Beugung 10/0/135°	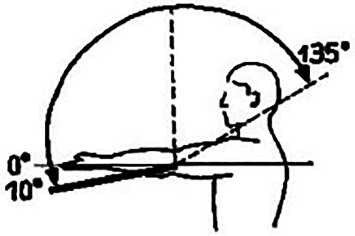
*Unterarmdrehung* Auswärts/einwärts 80–90/0/80–90°	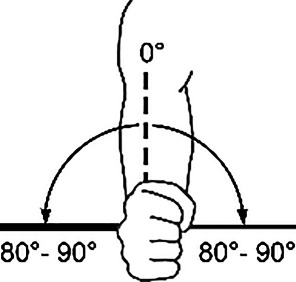
*Handgelenk* **(Nach eigenen Literaturrecherchen**[6]**)** Handrücken-/hohlhandwärts 60–80/0/60–80° Speichenwärts/ellenwärts 20–30/0/40–60°	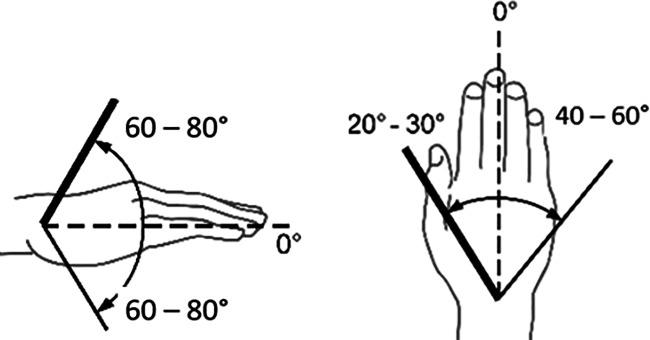
*Handgelenk* **(nach Messblättern der DGUV)** Handrücken-/hohlhandwärts 40–60/0/50–70° Speichenwärts/ellenwärts 20–30/0/30–40°	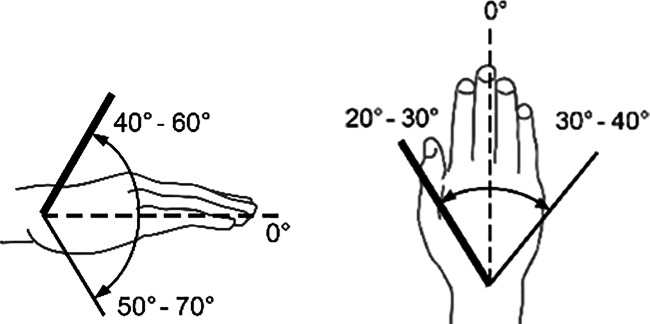
*Daumenabspreizung* In Handebene 50–70° Rechtwinklig dazu 50–70°	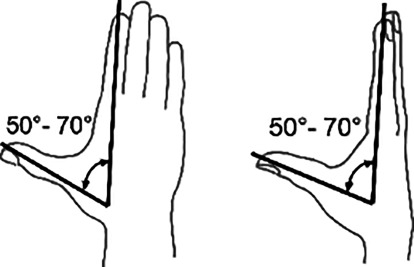
*Hüftgelenk* Streckung/Beugung 5–10/0/130°	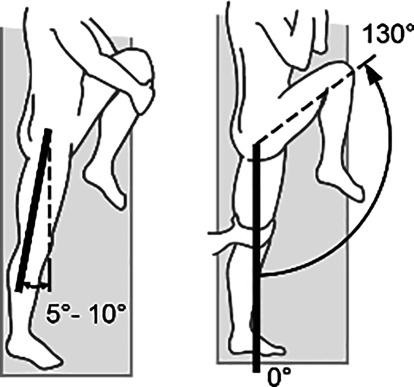
*Kniegelenk* Streckung/Beugung 5–10/0/130°	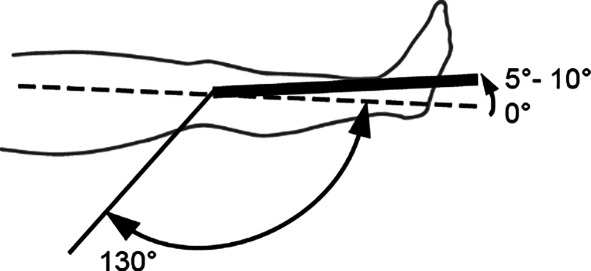
*Oberes Sprunggelenk* **(Nach eigenen Literaturrecherchen** [4]**)** Fußrücken-/fußsohlenwärts 10–20/0/40–55°	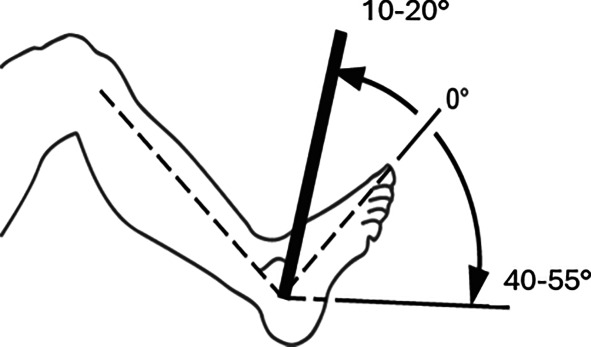
*Oberes Sprunggelenk* **(Nach Messblättern der DGUV)** Fußrücken-/fußsohlenwärts 20/0/40°	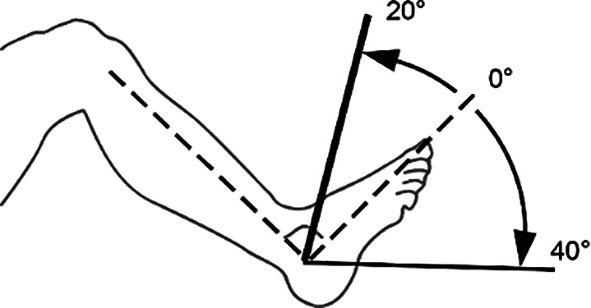

Skizzen mit freundlicher Genehmigung der DGUV, z. T. entnommen den von ihr zur Verfügung gestellten Messblättern [1], wobei die Werte am Hand- und am oberen Sprunggelenk (Zeilen 4 und 9) entsprechend den Literaturrecherchen zu diesen Bemessungsempfehlungen eigentlich angepasst werden müssten. Da die von den Autoren recherchierten Werte von der DGUV zwar als korrekt, aber in den Messblättern für nicht korrekturbedürftig angesehen werden, da nicht MdE-relevant, ergibt sich eine Abweichung der Eckwerte im Vergleich zur Erstpublikation. Es werden also die Normbeweglichkeiten der Messblätter verwendet (Zeilen 5 und 10), da diese allgemein Anerkennung finden

## Gliedertaxe – obere Gliedmaßen

### A – Verlustwerte (Tab. 2, Abb. 4 und 5)

#### AUB-Musterbedingungen (Deutschland, Stand 2020).

- Arm: 70 %
- Arm bis oberhalb des Ellenbogengelenks: 65 %
- Arm unterhalb des Ellenbogengelenks: 60 %
- Hand: 55 %
- Daumen: 20 %
- Zeigefinger: 10 %
- Anderer Finger: 5 %

**Tab. 2:** Verlustwerte von Daumenstrahl und Langfingern (Abb. 4 und 5)

Verlustwerte von Daumen- bzw. Fingergliedern	
Daumen	20/20 D
Daumenendglied	12/20 D
Daumen und 1. Mittelhandknochen	10/20 H
Langfinger	20/20 Fi
Langfingerendglied	8/20 Fi
Langfingermittel- und Langfingerendglied	14/20 Fi

**Abb. 4:**
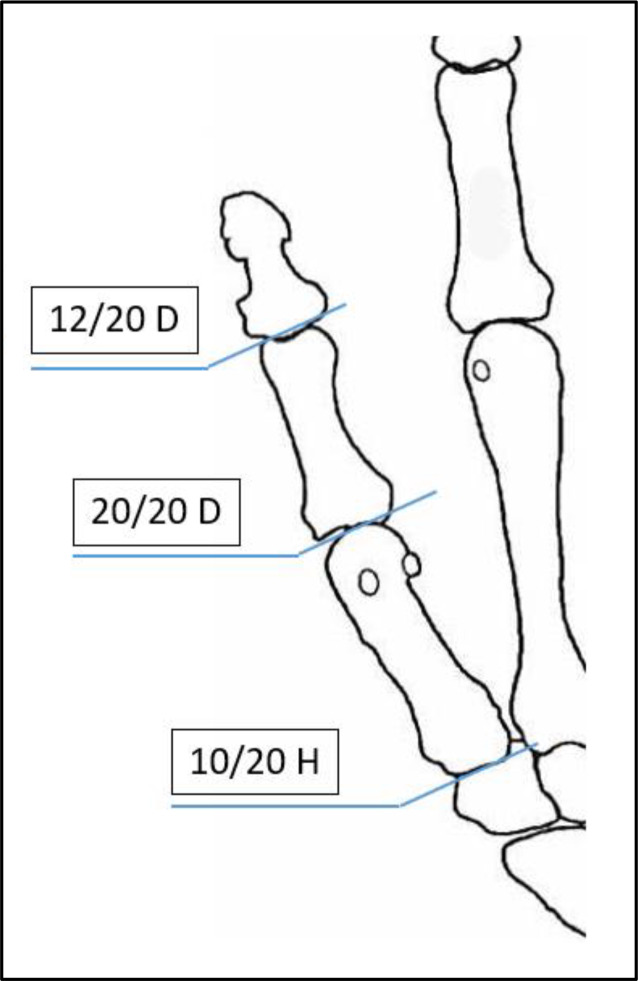
Verlustwerte am Daumenstrahl (Tab. 2)

**Abb. 5:**
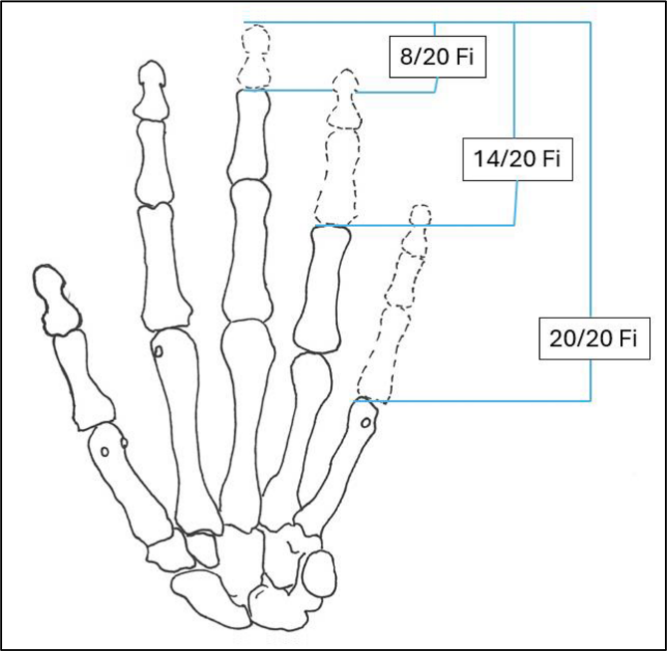
Verlustwerte Langfinger (Tab. 2)

### B – Versteifungswerte (Tab. 3, 4 und 5; Abb. 6, 7, 8, 9, 10, 11, 12 und 13)

Es gilt der Grundsatz: **Je stammnäher die Versteifung, desto ausgeprägter ist die Funktionsbeeinträchtigung.**

**Tab. 3:** Versteifungswerte in funktionsgünstiger Stellung an den oberen Gliedmaßen (isoliert) – Abb. 6, 7, 8, 9, 10, 11, 12 und 13

Versteifungswerte in funktionsgünstiger Stellung (isoliert ein Gelenk)	
Schultergelenk (Abb. 6)	8/20 A
Ellenbogengelenk bei freier Unterarmdrehung (Abb. 7)	6/20 A
Unterarmdrehung, aufgehoben in Auswärtsdrehung (Abb. 8a)^1^	7/20 H
Unterarmdrehung, aufgehoben in Neutral-0-Stellung (Abb. 8b)^1^	6/20 H
Unterarmdrehung, aufgehoben in Einwärtsdrehung (Abb. 8c)^1^	5/20 H
Handgelenk (Abb. 9)	5/20 H
Daumensattel- und Daumengrundgelenk (Abb. 10 und 11)	6/20 H^2^
Daumensattelgelenk (Abb. 10 und 11)	4/20 H^3^
Daumengrund- und Daumenendgelenk (Abb. 10 und 11)	12/20 D^4^
Daumenendgelenk (Abb. 10 und 11)	6/20 D
Daumengrundgelenk (Abb. 10 und 11)	4/20 D
Langfingergrund‑, Langfingermittel- und Langfingerendgelenk (Abb. 12 und 13)	18/20 Fi
Langfingergrund- und Langfingermittelgelenk (Abb. 12 und 13)	14/20 Fi
Langfingermittel- und Langfingerendgelenk (Abb. 12 und 13)	10/20 Fi
Langfingergrundgelenk (Abb. 12 und 13)	8/20 Fi
Langfingermittelgelenk (Abb. 12 und 13)	6/20 Fi
Langfingerendgelenk (Abb. 12 und 13)	4/20 Fi

^1^Die isolierte Aufhebung oder Einschränkung der Unterarmdrehung ist wegen der aus ihr folgenden Funktionsbeeinträchtigung der Handfunktion nach Handwert zu bemessen. Die *Aufhebung* der Unterarmdrehung tritt praktisch nie auf, wird regelhaft operativ korrigiert. Trotzdem werden Eckwerte benannt, um die z. T. extreme Funktionsbeeinträchtigung als Vergleichswert für andere Handfunktionsbeeinträchtigungen heranziehen zu können. Diesbezüglich wurde auch beim Vergleich mit der Erstpublikation eine deutliche Erhöhung der Eckwerte vorgenommen, da u. a. Ausgleichsbewegungen mit inkludiert waren

^2^Invaliditätswert für die Versteifung von Daumengrund- und Sattelgelenk im Vergleich zur Erstpublikation geändert, da die Funktionsbeeinträchtigung geringer ist als die Daumenamputation, aber schlechter als Handgelenkversteifung

^3^Wert wurde von ursprünglich 6 auf 4/20 reduziert, da die Versteifung des Handgelenks in funktionsgünstiger Stellung zu größeren Funktionsbeeinträchtigungen führt. Die Bemessung erfolgt nach Hand- und nicht Daumenwert, da globale Handfunktionen betroffen sind, die sich allein im Daumenwert nicht abbilden lassen

^4^Hier kommt es bei subsumierender Betrachtung zu einer Potenzierung des Invaliditätswertes über die reine Addition hinaus, da grundlegende Greiffunktionen beeinträchtigt sind

**Tab. 4:** Versteifungswerte in funktionsgünstiger Stellung an den oberen Gliedmaßen (kombiniert) nach Armwert

Auswirkung der Aufhebung der Unterarmdrehfähigkeit bei Ellenbogenversteifung in funktionsgünstiger Stellung auf den Armwert			
Ellenbogengelenk	Handgelenk	Unterarmdrehung aufgehoben in	Invalidität
Versteift	Frei	Auswärtsdrehung	12/20 A
Versteift	Frei	Neutral-0-Stellung	8/20 A^1^
Versteift	Frei	Einwärtsdrehung	7/20 A

^1^Erhöhung des Werts, da in der Erstpublikation Ausgleichsbewegungen eingerechnet wurden

**Tab. 5:** Versteifungswerte in funktionsgünstiger Stellung an den oberen Gliedmaßen (kombiniert) nach Handwert

Auswirkung der Aufhebung der Unterarmdrehfähigkeit bei Handgelenkversteifung in funktionsgünstiger Stellung auf den Handwert			
Ellenbogengelenk	Handgelenk	Unterarmdrehung aufgehoben in	Invalidität
Frei	Versteift	Auswärtsdrehung	11/20 H
Frei	Versteift	Neutral-0-Stellung	9/20 H^1^
Frei	Versteift	Einwärtsdrehung	8/20 H

^1^Erhöhung des Werts, da in der Erstpublikation Ausgleichsbewegungen eingerechnet wurden

**Abb. 6:**
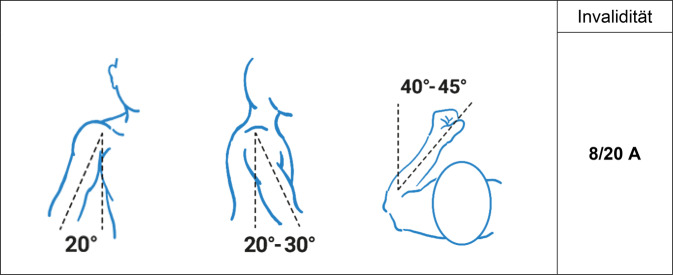
Versteifung des Schultergelenks in funktionsgünstiger Stellung (Tab. 3)

**Abb. 7:**
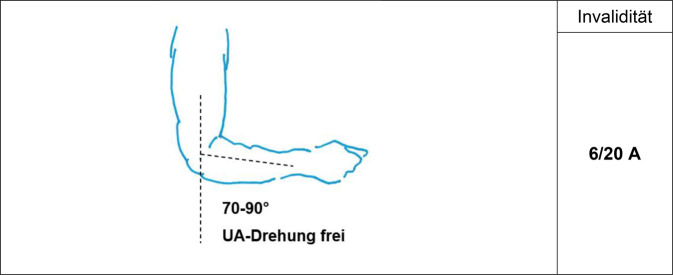
Versteifung des Ellenbogengelenks in funktionsgünstiger Stellung (UA-Drehung frei; Tab. 3)

**Abb. 8:**
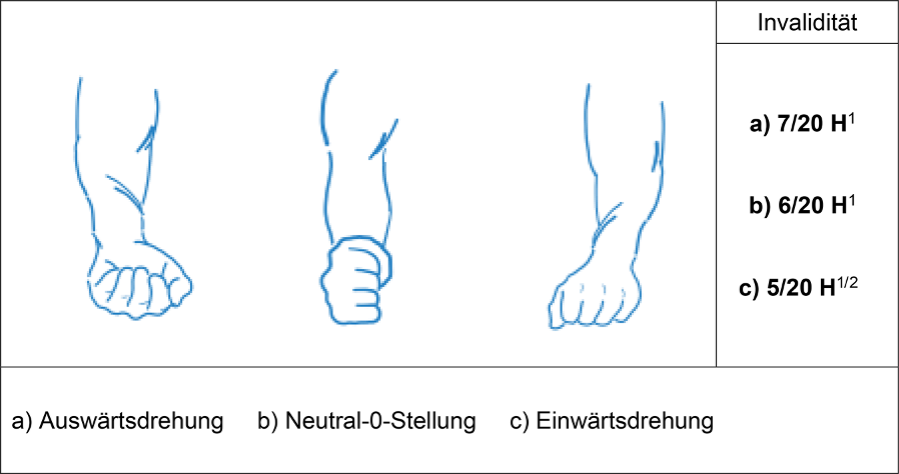
Aufhebung der Unterarmdrehung in verschiedenen Stellungen (Tab. 3). ^1^Die isolierte Aufhebung oder Einschränkung der Unterarmdrehung ist wegen der aus ihr folgenden Funktionsbeeinträchtigung der Handfunktion nach Handwert zu bemessen. Die Aufhebung der Unterarmdrehung tritt praktisch nie auf, wird regelhaft operativ korrigiert. Trotzdem werden Eckwerte benannt, um die z. T. extreme Funktionsbeeinträchtigung als Vergleichswert für andere Handfunktionsbeeinträchtigungen heranziehen zu können. Diesbezüglich wurde auch beim Vergleich mit der Erstpublikation eine deutliche Erhöhung der Eckwerte vorgenommen, da u. a. Ausgleichsbewegungen mit inkludiert waren. ^2^Diese Funktionsbeeinträchtigung entspricht der bei Versteifung des Handgelenks in funktionsgünstiger Stellung verbleibender Funktionsbeeinträchtigung

**Abb. 9:**
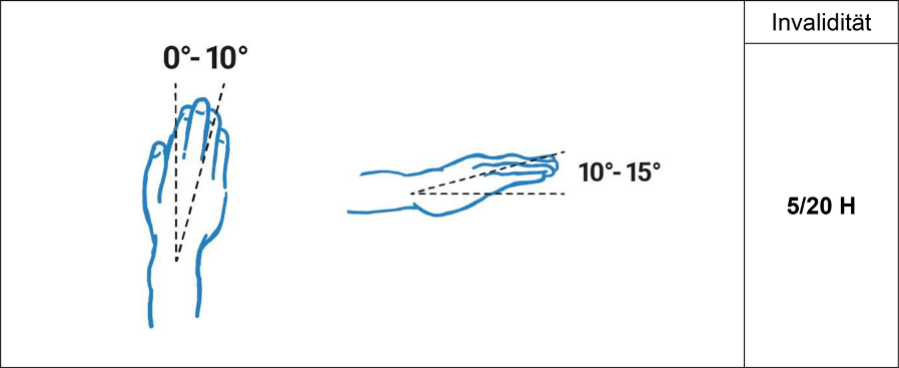
Versteifung des Handgelenks in funktionsgünstiger Stellung (Tab. 3)

**Abb. 10:**
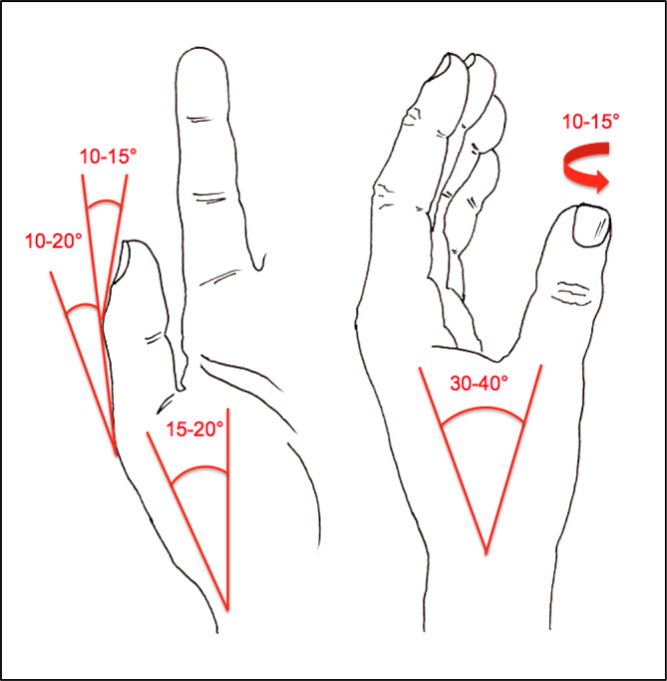
Versteifung des Daumenstrahls in funktionsgünstiger Stellung (Tab. 3)

**Abb. 11:**
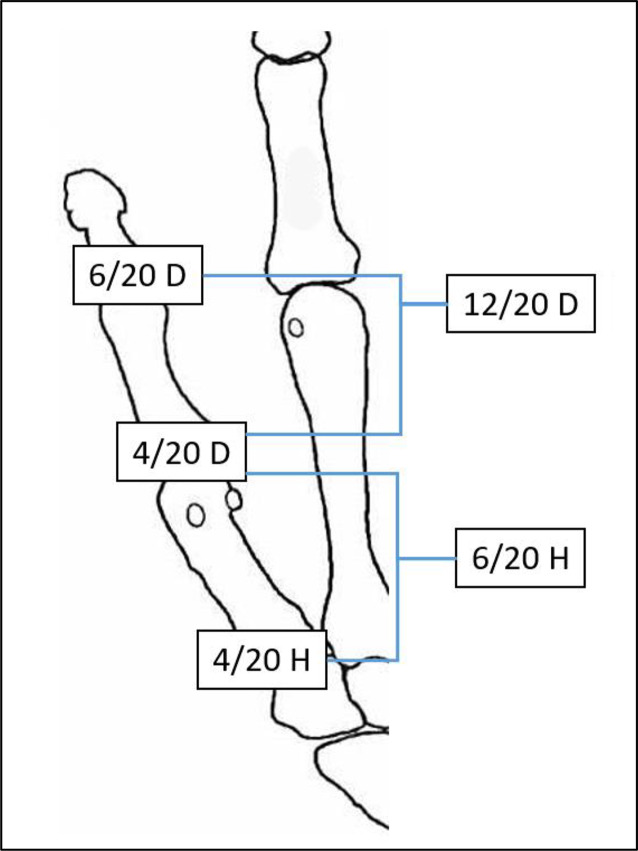
Invaliditätswerte für verschiedene Daumenstrahlgelenkversteifungen in funktionsgünstiger Stellung isoliert und kombiniert (Tab. 3 mit Fußnoten 2–4)

**Abb. 12:**
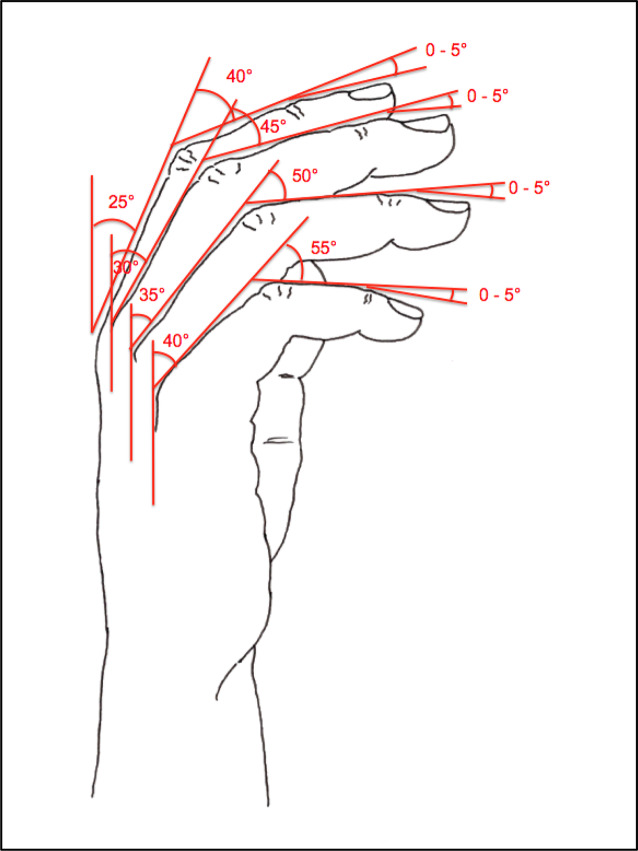
Versteifungsstellung der Langfingergelenke in funktionsgünstiger Stellung

**Abb. 13:**
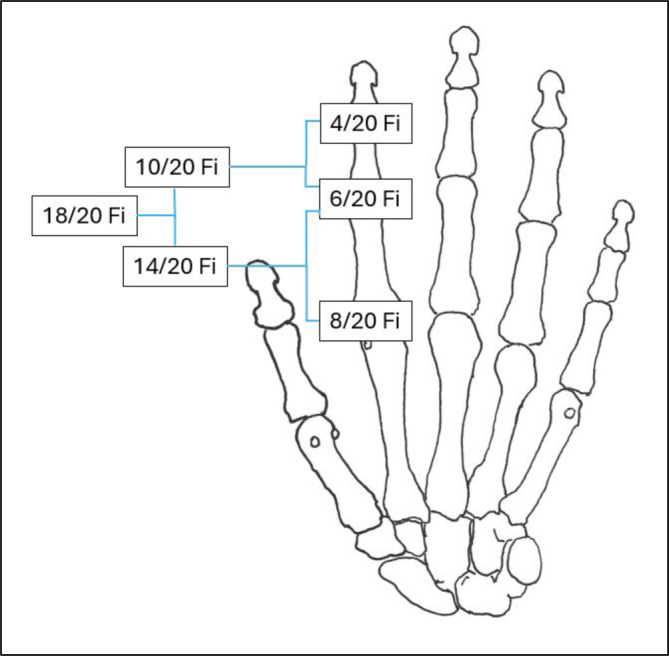
Invaliditätswerte für Langfingergelenkversteifungen in funktionsgünstiger Stellung (isoliert und kombiniert; Tab. 3)

Ist das Kugelgelenk Schulter versteift, kann die Hand die meisten Orte für ihren Gebrauch nicht mehr erreichen; ein Gewinn der freien Beweglichkeit des Scharniergelenkes Ellenbogen resultiert lediglich in einer Ebene. Umgekehrt kann die Hand mit versteiftem Ellenbogenscharniergelenk und freiem Schulterkugelgelenk unzählige Orte mehr im sog. Konfigurationsraum erreichen.

### C – Werte für Funktionsbeeinträchtigungen (Tab. 6, 7, 8, 9 und 10; Abb. 14)

Die funktionell bedeutendste Bewegungsebene ist die in Neutral-0-Blickrichtung. Insofern werden die Eckwerte für Einschränkungen in diesen Ebenen angegeben. Liegt zusätzlich eine *belangvolle* Funktionsbeeinträchtigung in einer anderen Ebene vor, muss der Sachverständige plausibel klären, ob daraus über die regelhafte Kombination derartiger Gelenkfunktionsbeeinträchtigungen ggf. zusätzliche Einschränkungen der Funktion vorhanden sind.

**Tab. 6:** Funktionsbeeinträchtigungen im Schultergelenk

Funktionsbeeinträchtigungen im Schultergelenk^1^ (Abb. 14)	
Einschränkung der Vorhebung bis 120°	1/20 A
Einschränkung der Vorhebung bis 90°	3/20 A
Einschränkung der Vorhebung bis 60°	5/20 A
Einschränkung der Vorhebung bis 30°	7/20 A
Aufhebung der Rückführfähigkeit mit Unmöglichkeit von Schürzen- und Gesäßgriff	2/20 A
Persistierende Schultereckgelenkinstabilität Rockwood 2 oder höher, je nach individuellem Funktionsdefizit im Vergleich zu anderen Eckwerten von Schulterfunktionsbeeinträchtigungen	1‑2/20 A
Verformung/Subluxation im Schlüsselbein‑/Brustbeingelenk mit klinischer Symptomatik	1/20 A
Vollständiger Funktionsverlust der langen Bizepssehne mit Kraftminderung bei der Seitwärtshebung des Armes im Schultergelenk, bei der Beugung im Ellenbogengelenk und bei der Auswärtsdrehung des Unterarmes^2^	1/20 A
Vollständiger Funktionsverlust der körperfernen Bizepssehne mit Einschränkung der Beugung im Ellenbogengelenk und der Unterarmdrehung^3^	2/20 A

^1^Soll die Schulterrechtsprechung des BGH vom 01.04.2015 Anwendung finden, so ist die Invalidität außerhalb der Gliedertaxe zu bemessen (Tab. 22). Die Instanzgerichte setzen die Rechtsprechung des BGH jedoch meist nicht um

^2^Der alleinige Defekt der Sehne rechtfertigt keine Invaliditätsbemessung, würde einer diagnosenassoziierten Invalidität entsprechen. Es ist also unbedingt darauf zu achten, dass die genannten Funktionsbeeinträchtigungen auch tatsächlich belegt sind

**Tab. 7:** Funktionsbeeinträchtigungen im Ellenbogengelenk bei freier Unterarmdrehung

Funktionsbeeinträchtigungen im Ellenbogengelenk bei freier Unterarmdrehung		
**Beugung bis 120°**	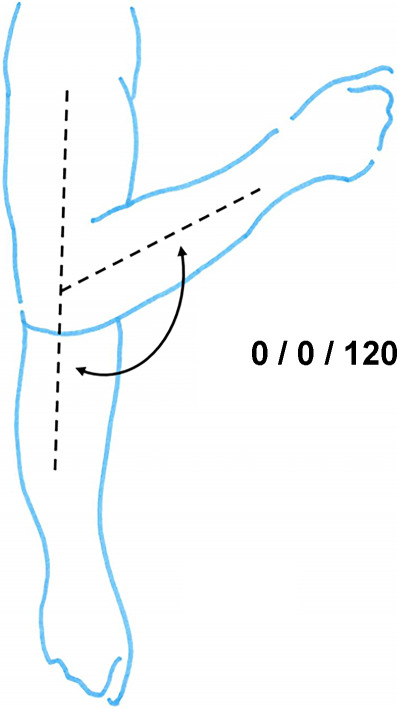	**1/20 A**
**Beugung über 120° und Streckdefizit von 20 bis 30°**	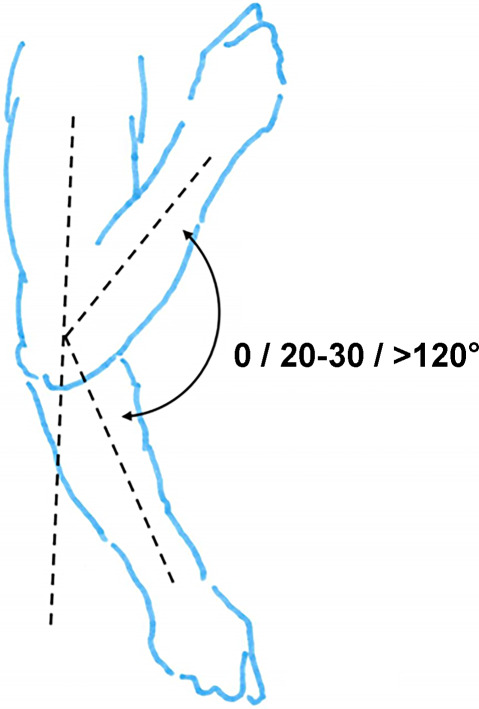	**1/20 A**
**Beugung bis 120° und Streckdefizit von 20 bis 30°**	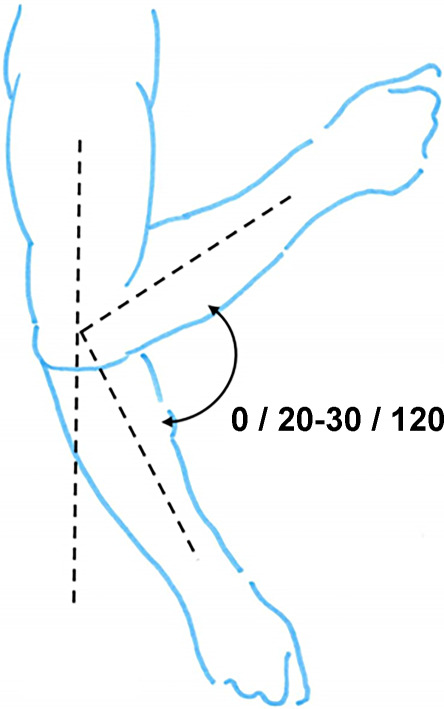	**2/20A**
**Beugung bis 90°**	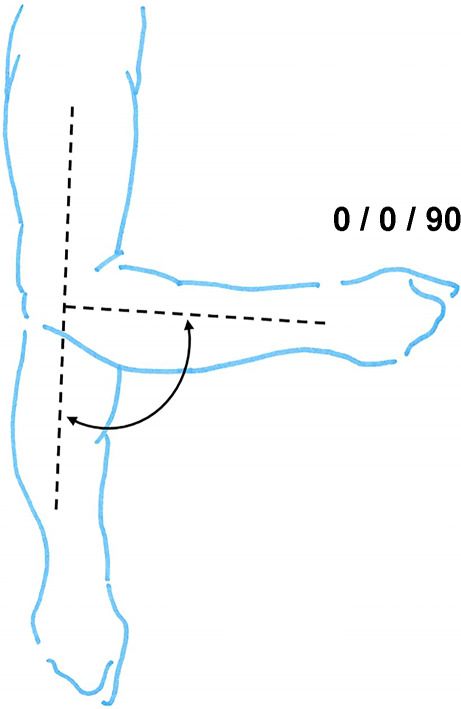	**4/20 A**
**Beugung bis 90° und Streckdefizit von 20 bis 30°**	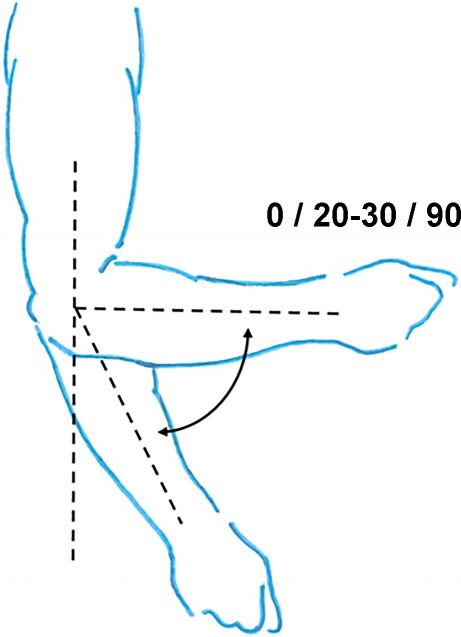	**5/20A**

**Tab. 8:** Einschränkung der Unterarmdrehung

Einschränkung der Unterarmdrehung auswärts/einwärts^1^		
**45–0–45**	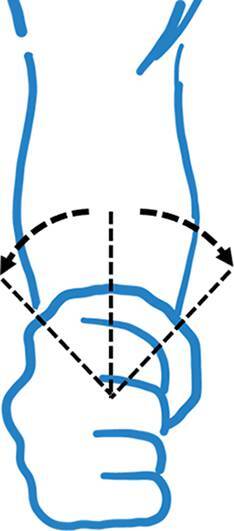	**2/20 H**^**2**^
**90–0–45**	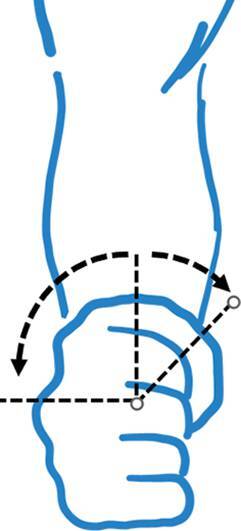	**2/20 H**^**2**^
**45–0–90**	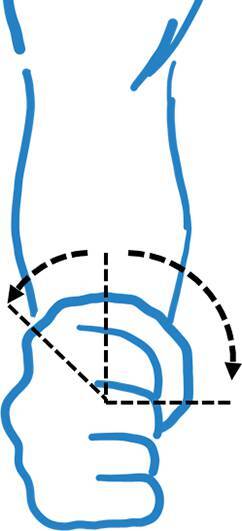	**1/20 H**^**2**^

^1^Im Vergleich zur Erstpublikation neu aufgenommen

^2^Die isolierte Aufhebung oder Einschränkung der Unterarmdrehung ist wegen der aus ihr folgenden Funktionsbeeinträchtigung der Handfunktion nach Handwert zu bemessen. Die *Aufhebung* der Unterarmdrehung tritt praktisch nie auf, wird regelhaft operativ korrigiert. Trotzdem werden Eckwerte benannt, um die z. T. extreme Funktionsbeeinträchtigung als Vergleichswert für andere Handfunktionsbeeinträchtigungen heranziehen zu können. Diesbezüglich wurde auch beim Vergleich mit der Erstpublikation eine deutliche Erhöhung der Eckwerte vorgenommen, da u. a. Ausgleichsbewegungen mit inkludiert waren

**Tab. 9:** Funktionsbeeinträchtigungen im Handgelenk bei freier Unterarmdrehung

Funktionsbeeinträchtigungen im Handgelenk	
Konzentrische Bewegungseinschränkung um drei Viertel der Norm	4/20 H
Konzentrische Bewegungseinschränkung um zwei Viertel der Norm	3/20 H
Konzentrische Bewegungseinschränkung um ein Viertel der Norm	2/20 H

**Tab. 10:** Funktionsbeeinträchtigungen Daumen und Finger

Daumen- und Fingerfunktionsbeeinträchtigungen	
Instabilität des Daumengrundgelenks nach „Skidaumen“	2/20 D
Instabilität des Daumengrundgelenks mit Einschränkung der Gegenüberstellfähigkeit des Daumens nach „Skidaumen“	4/20 D
Fehlende aktive Streckbarkeit am Endgelenk eines Langfingers bei z. B. Defekt der Strecksehne	2/20 Fi

**Abb. 14:**
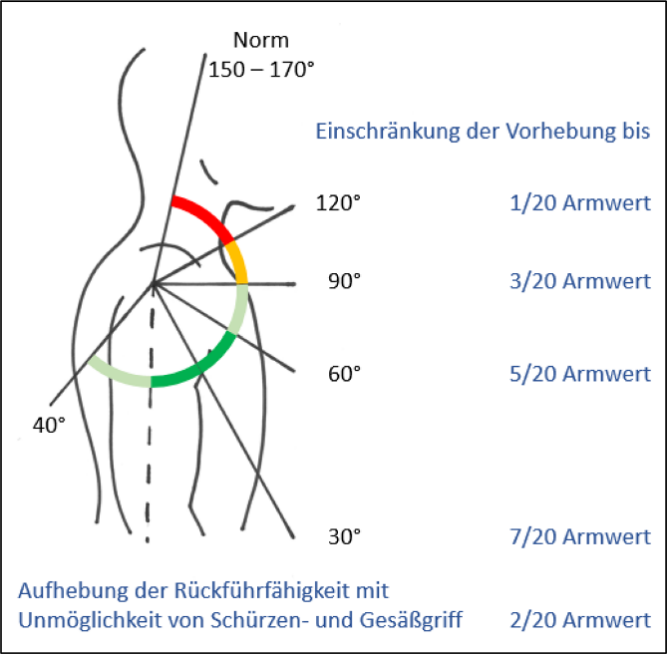
Bewegungseinschränkung des Schultergelenks innerhalb der Gliedertaxe (Tab. 6). Die hier beschriebenen Funktionseinschränkungen sind regelhaft mit Rotationseinschränkungen und Minderung der Abhebefähigkeit seitwärts vergesellschaftet und rechtfertigen ohne plausible Begründung keine Erhöhung. Erläuterung des Farbschemas: *dunkelgrün* funktionell von herausragender Bedeutung, *hellgrün* funktionell von großer Bedeutung, *gelb* funktionell von weniger großer Bedeutung, *rot* funktionell von untergeordneter Bedeutung

## Gliedertaxe – untere Gliedmaßen

### A – Verlustwerte (Tab. 11; Abb. 15)

#### AUB-Musterbedingungen (Deutschland, Stand 2020).

- Bein über der Mitte des Oberschenkels: 70 %
- Bein bis zur Mitte des Oberschenkels: 60 %
- Bein bis unterhalb des Knies: 50 %
- Bein bis zur Mitte des Unterschenkels: 45 %
- Fuß: 40 %
- Große Zehe: 5 %
- Andere Zehe: 2 %

**Tab. 11:** Verlustwerte an den unteren Gliedmaßen (Abb. 15)

Verlustwerte von Fußteilen	
Chopart-Amputation (Amputation in der sog. Chopart-Gelenklinie (lediglich Rückfuß erhalten mit Sprung- und Fersenbein)	14/20 F
Lisfranc-Amputation (Amputation in der sog. Lisfranc-Gelenklinie zwischen Fußwurzel und Mittelfuß)	10/20 F
Sharp-Amputation (Mittelfuß teilweise erhalten)	7/20 F

**Abb. 15:**
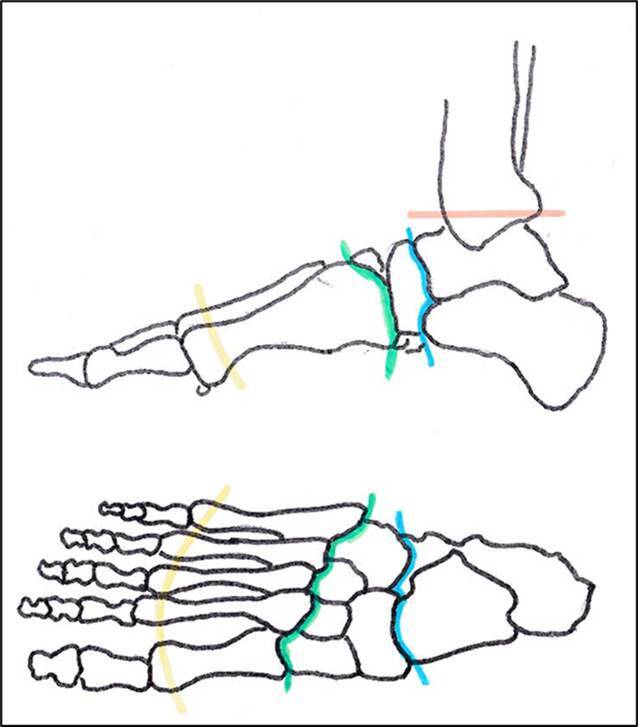
Verlustwerte am Fuß (Tab. 11). *Orange: *Syme-Amputation entspr. Fußverlust*, hellblau: *Chopart-Amputation (der Eckwert bezieht sich auf eine gebrauchsgünstige Stellung, umfasst also nicht den häufigen Fall, dass es durch Zug der Achillessehne bei ungenügender Refixation der Tibialis-anterior-Sehne zu einer ungünstigen Spitzfußstellung kommt, die trotz orthetischer Versorgung eine Belastung des Fußes unmöglich macht), *grün: *Lisfranc-Amputation, *gelb: *Sharp-Amputation (Mittelfuß teilweise erhalten)

### B – Versteifungswerte (Tab. 12 und 13; Abb. 16, 17, 18 und 19)

Es gilt der Grundsatz: **Je stammnäher die Versteifung, desto ausgeprägter ist die Funktionsbeeinträchtigung.**

**Tab. 12:** Versteifungswerte der unteren Extremität in funktionsgünstiger Stellung

Versteifungswerte in funktionsgünstiger Stellung	
Hüftgelenk (Abb. 16)	10/20 B
Kniegelenk (Abb. 17)	8/20 B
Oberes Sprunggelenk (Abb. 18)	6/20 F
Unteres Sprunggelenk^1^	4/20 F
Oberes und unteres Sprunggelenk	9/20 F
Großzehengrundgelenk^2^ (Abb. 19)	8/20 Gz
Grundgelenk andere Zehe	6/20 Z

^1^Hinteres und vorderes unteres Sprunggelenk werden als eine funktionelle Einheit betrachtet

^2^Die ursprünglich postulierte funktionsgünstige Großzehenversteifung im Grundgelenk in Dorsalextension von 20-25° wurde wieder verlassen, da sie in Relation zur Fußauftrittsebene des belasteten Fußes schwer messbar ist. Es wurde konsentiert, dass man am belasteten Fuß beurteilt, ob die Großzehe bei Flexion im Endgelenk suffizienten Bodenkontakt hat. Dies mindert auch die Schwierigkeit der Beurteilung der Stellung bei Fußfehlformen (Hohl- oder Plattfuß)

**Tab. 13:** Versteifungswerte der Zehen in funktionsungünstiger Stellung

Versteifungswerte der Zehen in funktions*un*günstiger Stellung	
Großzehengrundgelenk in Neutral-0-Stellung	12/20 Gz
Großzehengrundgelenk in Beugestellung	20/20 Gz
Grundgelenk andere Zehe in funktions*un*günstiger Stellung	10/20 Z

**Abb. 16:**
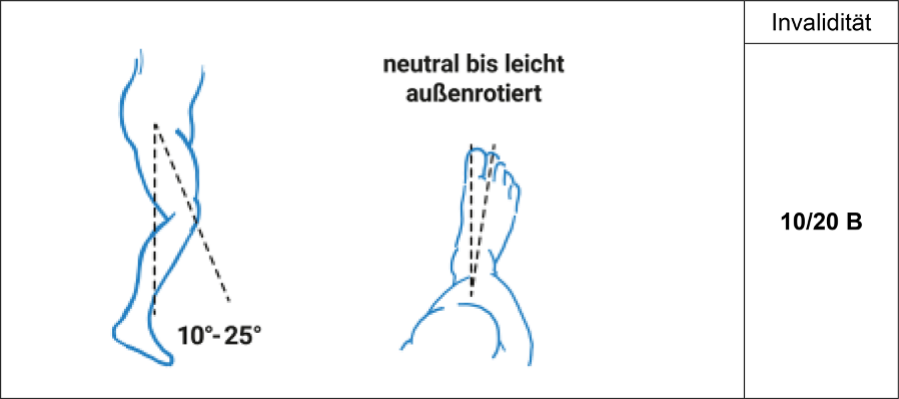
Versteifung des Hüftgelenks in funktionsgünstiger Stellung (Tab. 12)

**Abb. 17:**
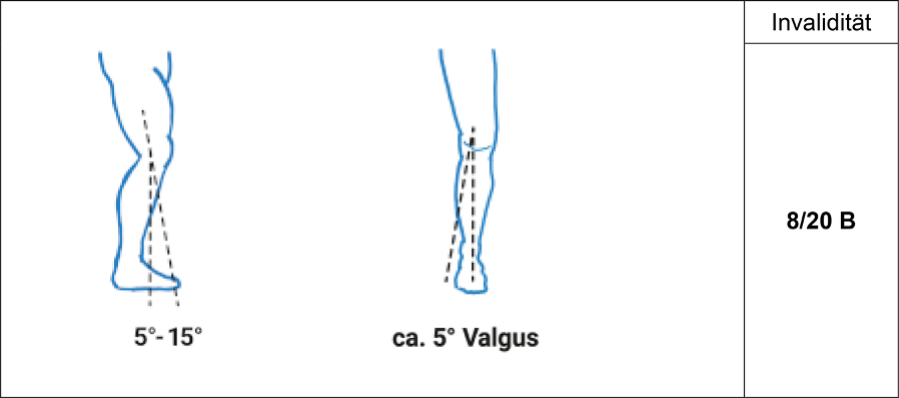
Versteifung des Kniegelenks in funktionsgünstiger Stellung (Tab. 12)

**Abb. 18:**
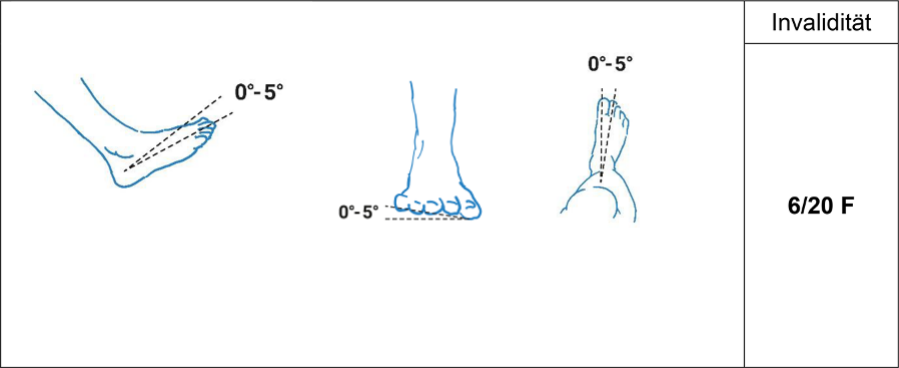
Versteifung des oberen Sprunggelenks in funktionsgünstiger Stellung (Tab. 12)

**Abb. 19:**
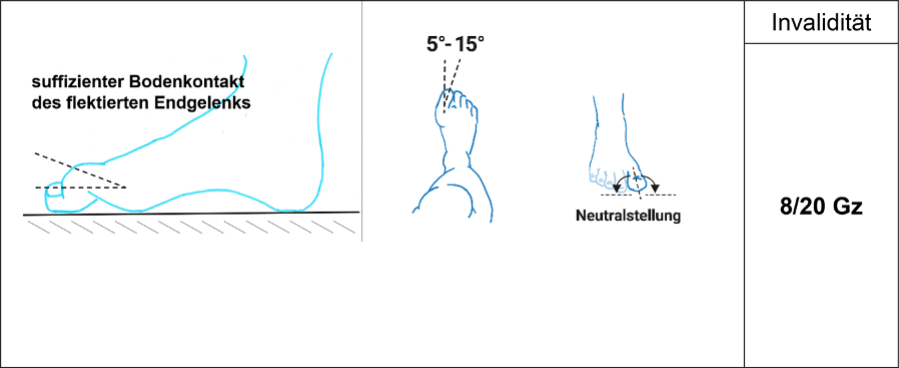
Versteifung der Großzehe in funktionsgünstiger Stellung (Tab. 12)

### C – Werte für Funktionsbeeinträchtigungen (Tab. 14, 15, 16, 17 und 18)

Die funktionell bedeutendsten Bewegungsebenen sind die in Fortbewegungsrichtung. Insofern werden die Eckwerte für Einschränkungen in diesen Ebenen angegeben. Liegt zusätzlich eine *belangvolle* Funktionsbeeinträchtigung in einer anderen Ebene vor, muss der Sachverständige plausibel klären, ob daraus über die regelhafte Kombination derartiger Gelenkfunktionsbeeinträchtigungen ggf. zusätzliche Einschränkungen der Funktion vorhanden sind.

**Tab. 14:** Funktionsbeeinträchtigungen im Hüftgelenk

Funktionsbeeinträchtigungen im Hüftgelenk	
Streckdefizit	
> 5° bis ≤ 10°	1/20 B
Beugung	
Bis 120°	1/20 B
Bis 90°	2/20 B
Bis 60°	4/20 B
Bis 30°	8/20 B

**Tab. 15:** Funktionsbeeinträchtigungen im Kniegelenk

Funktionsbeeinträchtigungen im Kniegelenk	
*Streckdefizit*	
> 5° bis ≤ 10°	1/20 B
> 10°bis ≤ 15°	2/20 B
> 15° bis ≤ 20°	3/20 B
> 20°	Je nach Ausmaß, mindestens jedoch 4/20 B
*Beugung*	
Bis 90°	1/20 B
Bis 60°	4/20 B
Bis 30°	7/20 B
*Instabilität*	
Leichtgradig eindimensional^1^	1/20 B
Leichtgradig zweidimensional^1^	3/20 B
Mittelgradig eindimensional^1^	3/20 B
Mittelgradig zweidimensional^1^	6/20 B
Hochgradig eindimensional^1^	5/20 B
Hochgradig zweidimensional^1^	10/20 B

^1^Leicht-, mittel- und hochgradig werden nach der klinischen Bandnachgiebigkeit definiert wie folgt: leichtgradig: > 3 mm bis ≤ 5 mm; mittelgradig: > 5 mm bis ≤ 10 mm; hochgradig: > 10 mm

**Tab. 16:** Funktionsbeeinträchtigungen der Sprunggelenke

Funktionsbeeinträchtigungen der Sprunggelenke	
*Oberes Sprunggelenk: Bewegungseinschränkung (fußrücken-/fußsohlenwärts) auf Werte von*	
10/0/30	2/20 F^1^
10/0/20	3/20 F^1^
0/0/30	3/20 F
0/0/20	4/20 F
0/0/10	5/20 F
0/10/x*	10/20 F
0/> 10/x*	12/20 F
*x deshalb, weil die Restbeugefähigkeit bei Spitzfuß relativ unerheblich ist, bei Erhalt der Restbeugefähigkeit also kein relevanter Funktionsgewinn erzielt wird	
*Unteres Sprunggelenk: Bewegungseinschränkung um*	
Ein Drittel der Norm	2/20 F
Zwei Drittel der Norm	3/20 F
Kombinierte Funktionsbeeinträchtigungen von oberem und unterem Sprunggelenk sind nicht additiv, sondern subsumierend zu bewerten. Als ein Vergleichswert gilt dabei die Versteifung von oberem und unterem Sprunggelenk mit 9/20 F	

^1^Wert im Vergleich zur Erstpublikation erhöht, da zur Vereinheitlichung die Normbeweglichkeit nach derzeit Anwendung findenden Messblättern der DGUV zugrunde gelegt wird (Tab. 1, *Anmerkungen*)

**Tab. 17:** Funktionsbeeinträchtigungen durch isolierte Längenabweichungen

Funktionsbeeinträchtigungen durch (isolierte) Längenabweichungen^1^	
> 1 cm bis ≤ 2 cm	1/20 B
> 2 cm bis ≤ 3 cm	2/20 B
> 3 cm bis ≤ 5 cm	3/20 B
Unfallbedingte Längendifferenzen > 5 cm bedürfen einer ganz individuellen Betrachtung, da regelhaft andere Verletzungsfolgen im Vordergrund stehen dürften	

^1^Längen- und Achsabweichungen treten selten isoliert auf und sind regelhaft subsumierend in der „Gesamt“-Invalidität zu berücksichtigen

**Tab. 18:** Funktionsbeeinträchtigungen durch isolierte Achsabweichungen

Funktionsbeeinträchtigungen durch (isolierte) Achsabweichungen^1^	
> 5° bis ≤ 10°	1/20 B
> 10° bis ≤ 20°	2/20 B
Unfallbedingte Achsabweichungen > 20° bedürfen einer ganz individuellen Betrachtung, da regelhaft andere Verletzungsfolgen im Vordergrund stehen dürften	

^1^Längen- und Achsabweichungen treten selten isoliert auf und sind regelhaft subsumierend in der „Gesamt“-Invalidität zu berücksichtigen

### Thrombosefolgen und unfallbedingte Lymphödeme

Diese sind in der Regel durch einen internistischen/angiologischen Gutachter unter Beachtung der Leitlinien zu Diagnostik und Therapie der Venenthrombose und Lungenembolie zu beurteilen. Insbesondere bei einem eindeutig zu definierenden postthrombotischen Syndrom geht es v. a. um den doppler-/duplexsonographischen Befund und die Bemessung eines begleitenden Ödems. Eine blutgerinnungshemmende Therapie ist außerhalb der Gliedertaxe zu bemessen. Eine Umfangsvermehrung sagt noch nichts aus über die (wiedererlangte) Durchgängigkeit des Gefäßsystems und kann nicht alleinige Grundlage einer Invaliditätsbemessung sein.

*Ist die Durchgängigkeit der Gefäße nach einer Thrombose sonographisch belegt, liegt keine Klappeninsuffizienz vor, und beträgt die Umfangsdifferenz weniger als 2* *cm, kann auf eine Zusatzdiagnostik/Zusatzbegutachtung verzichtet werden, da dann keine Invalidität bemessen werden kann.*

Empfehlungen werden aktuell mit den internistischen Fachkollegen diskutiert und werden nach Konsentierung unter invaliditaet-online.de abrufbar sein.

### Unfallbedingte Arthrosen

Für die Beurteilung von Funktionsbeeinträchtigungen durch eine unfallbedingte Arthrose ist – wie grundsätzlich – der Zeitpunkt der Erstbemessung (12 bzw. 15 Monate nach Unfall) maßgeblich. Auf diesen Zeitpunkt müssen der Istzustand und dessen Prognose bezogen werden. Kommen zu diesem Zeitpunkt umformende Gelenkveränderungen bildgebend nicht zur Darstellung, kann die Möglichkeit negativer Veränderungen einer Prognose nicht zugrunde gelegt werden. Kommen sie aber bildgebend zur Darstellung, ist die Frage der Relevanz in Bezug auf die Prognose zu stellen, denn selbst bildgebend gesicherte Arthrosen müssen nicht zwangsläufig auch mit einer invaliditätsrelevanten Verschlechterung der Gelenkfunktion verknüpft sein. Nur wenn also zum Zeitpunkt der Erstbemessung eine unfallbedingte Arthrose bildgebend gesichert ist und daraus resultierende Funktionseinschränkungen vorliegen, ist deren weitere Prognose zu beachten. Liegen demgegenüber zum Zeitpunkt der Erstbemessung funktionell nichtrelevante unfallbedingte Arthrosezeichen vor, so ist eine Neubemessung kurz vor Ablauf des vereinbarten Regulierungszeitpunkts zu veranlassen (regelhaft vor Ablauf des 3. Unfalljahres).

### Unfallbedingte Endoprothesen

Pauschalierte Endoprothesenzuschläge in Abhängigkeit vom Alter sind nicht zu rechtfertigen. Es ist gutachtlich eine Beurteilung der Gelenkfunktion vorzunehmen und dann zu berücksichtigen, dass der Endoprothesenträger allein durch die einliegende Prothese funktions-, leistungs- und belastungslimitiert ist. Der ärztliche Sachverständige muss also dazu Stellung nehmen, inwieweit prothesen-, material-, zugangs- und/oder instrumentierungsassoziierte Folgen neben z. B. der Störung der Propriozeption vorhanden sind. Weiter muss er beurteilen, ob allein durch das Vorhandensein der Endoprothese bestimmte Funktionen z. B. aus präventiven Gründen vermieden werden müssen. Diese Faktoren wirken sich invaliditätsrelevant auf die Prognosebeurteilung aus, was in der Regel eine Invalidität von mindestens 1/20 Extremitätenwert nach sich zieht.

## Invalidität außerhalb der Gliedertaxe

### Wirbelsäule

Die gutachtliche Bemessung von verbliebenen Funktionsbeeinträchtigungen am Achssystem bzw. nichtpaarigen Organen des Menschen stellt den ärztlichen Sachverständigen vor ganz besondere Herausforderungen. Der Sachverständige muss alle Einflussfaktoren auf das funktionelle Endergebnis, die sich bereits aus Art und Ausmaß der Erstgesundheitsschädigung, aber auch aus den unterschiedlichen Ausheilungsmöglichkeiten in Abhängigkeit von der betroffenen funktionellen Bewegungsregion der Wirbelsäule ergeben, kennen.

Die Invalidität ist nicht punkt-/prozentgenau zu beziffern. Außerdem sind auch nach langstreckigen Versteifungen im Bereich mehrerer funktioneller Bewegungsregionen kaum Funktionsstörungen, die eine Invaliditätsbemessung über 30 % rechtfertigen könnten, vorstellbar, sofern keine zusätzlichen neurologischen Ausfälle zu beachten sind. Dies bedürfte einer individuell sehr plausiblen Erklärung. Würde man nun einer Systematik der Abstufung der Invalidität in 5er-Schritten folgen, so zeigt die Erfahrung, dass damit die verschiedenen Funktionsbeeinträchtigungen nicht ausreichend abzubilden sind, also auch Bemessungen z. B. zwischen den Werten 5 und 10 zu diskutieren sind, also 2,5 usw. Das scheint zunächst ein Widerspruch zur fehlenden Möglichkeit einer punktgenauen Invaliditätsbemessung, bestimmt aber letztlich nur systematisch einen definierten Zwischenwert.

In der nachfolgenden Systematik finden die erheblichen funktionellen Unterschiede verschiedener Wirbelsäulenabschnitte Beachtung. Unfallbedingt verbliebene Formverbildungen oder Versteifungen können nicht losgelöst vom betroffenen Wirbelsäulenabschnitt beurteilt werden. Die Versteifung eines Bewegungssegments der Halswirbelsäule zieht andere Funktionsbeeinträchtigungen nach sich als die eines Bewegungssegments der Brustwirbelsäule oder wieder andere bei Betroffenheit des thorakolumbalen Übergangs (Brustwirbelsäulen-Lendenwirbelsäulen-Übergang). Unabdingbar ist auch die Beantwortung der Frage nach dem Vorliegen einer Störung des sagittalen (Ebene, die sich von oben nach unten sowie von hinten nach vorn im Körper erstreckt – teilt also den Körper in einen linken und rechten Anteil) Profils mit Abweichung desselben vom ehemals (vor dem Unfall) bestehenden Profil der Wirbelsäule (∆GDW).

Aufgrund der erheblichen anatomischen und biomechanischen Unterschiede innerhalb des Achsenskeletts ist eine Gliederung zielführend, die neben anatomischen und biomechanischen Gesichtspunkten auch die jeweiligen funktionellen Besonderheiten berücksichtigt. Aufgrund der differenten Anzahl von Spinalnervsegmenten an der Halswirbelsäule wird eine Nomenklatur verwendet, die sich an der knöchernen Struktur orientiert, nämlich dem Wirbel selbst. Auch wird bewusst von einem Wirbel (z. B. HW2 für 2. Halswirbel) und nicht einem Wirbelkörper (z. B. HWK2 für 2. Halswirbelkörper) gesprochen, da Letzterer nur einen Teil des Wirbels darstellt.

- *Region 1:* kraniozervikaler Übergang (Kopf-Hals-Übergang) und obere Halswirbelsäule (Okziput (Hinterhaupt) bis HW2)
  Die Kopf-Hals-Gelenke bilden zusammen mit der oberen Halswirbelsäule (Atlas und Axis) eine funktionell geschlossene Einheit [11]. Die HW1‑/HW2-Gelenke sind auf Rotation ausgelegt. Das Zapfengelenk des Dens axis (Zahnfortsatz des 2. Halswirbels) ermöglicht 20°- bis 30°-Rotation zu jeder Seite. Bis zu 70 % der Kopfdrehung erfolgen aus diesem unteren Kopfgelenk, der Rest aus der übrigen HWS.
- *Region 2:* subaxiale (unterhalb des 2. Halswirbels) Halswirbelsäule (HW2–HW5)
  Die subaxiale HWS ist der wesentliche Bereich für die Seitneigung sowie Beugung und Streckung der Halswirbelsäule. Für die Rotation spielen diese Bewegungssegmente, verglichen mit dem unteren Kopfgelenk (HW1/HW2), nur eine untergeordnete Rolle.
- *Region 3:* zervikothorakaler (Übergang der Hals- zur Brustwirbelsäule) Übergang (HW5–BW2)
  In dieser Junktionszone trifft die Halswirbelsäule mit ihrem hohen Grad an Mobilität und Flexibilität auf die durch den Brustkorb stabilisierte und rigidere Brustwirbelsäule. Ähnlich dem Übergang zwischen Brust- und Lendenwirbelsäule ist dieser Abschnitt bedeutenden Belastungen ausgesetzt und wird bei Unfällen daher häufiger verletzt.
- *Region 4:* Brustwirbelsäule (BW2–BW10)
  Im Vergleich mit der Hals- und Lendenwirbelsäule ist die segmentale Beweglichkeit der Brustwirbelsäule, insbesondere für Beugung/Streckung, mit einer Amplitude von in der Regel unter 5° gering. Aufgrund der höheren Anzahl an Bewegungssegmenten hat dieser Wirbelsäulenabschnitt dennoch Bedeutung für die Beweglichkeit der Wirbelsäule im Gesamten (Seitneigung und Rotation).
- *Region 5:* Brustwirbelsäulen-Lendenwirbelsäulen-Übergang (BW10–LW2)
  Dieser Übergangsbereich nimmt eine Sonderstellung innerhalb der Wirbelsäule ein. Mehr als die Hälfte aller Verletzungen der Brust- und Lendenwirbelsäule betreffen die Region zwischen BW10 und LW2. Verantwortlich hierfür sind der Wechsel von der Kyphose (nach rückenwärts verstärkte Krümmung) der Brust- in die Lordose (Krümmung der Wirbelsäule nach vorn) der Lendenwirbelsäule, der Wegfall der stabilisierenden Wirkung des Brustkorbes und die Änderung der Ausrichtung der Wirbelgelenke von einer vorwiegend frontalen Stellung im BWS-Bereich zu einer nahezu sagittalen Orientierung im LWS-Bereich, was mit einem sprunghaften Anstieg der Rotationssteifigkeit verbunden ist [13]. Aufgrund der großen Rotationsmöglichkeit kommt diesem Übergang funktionell besondere Bedeutung zu.
- *Region 6:* Lendenwirbelsäule und lumbosakraler (Übergang Lendenwirbelsäule zum Kreuzbein) Übergang (LW2–SW1)
  Aufgrund der fast sagittalen Ausrichtung der Wirbelgelenke sind in diesem Abschnitt nur minimale Rotationsbewegungen möglich. Die mittlere Bewegungsamplitude für Flexion/Extension steigt vom thorakolumbalen zum lumbosakralen Übergang sukzessive an. Mit einer Amplitude von durchschnittlich 20° für Extension/Flexion besitzt das Bewegungssegment LW5/SW1 die größte segmentale Beweglichkeit der gesamten Wirbelsäule in dieser Raumebene.

Ist die Wertigkeit der Bewegungsregion geklärt, muss der ärztliche Sachverständige zu folgenden Fragen Stellung nehmen:

- Bewegungs-/Entfaltungsstörung
- Anzahl der betroffenen Bewegungssegmente
- zugangs-, instrumentierungs- und/oder bandscheibenassoziierte Folgen
- Stabilität des/der Bewegungssegment(e) und/oder der Instrumentierung
- Anschlussinstabilität oder -überlastung
- Knöcherne Ausheilung/Versteifung
- Störung der sagittalen Balance (∆GDW ≥ 15-20°)
- Störung der frontalen Balance (Skoliosewinkel > 10°)
- Morbidität durch Fremdmaterial und/oder Entnahmemorbidität
- Weitere klinische oder bildgebende Pathologika
- Neurologische Folgen (regelhaft separat zu bewerten).

Bezüglich weiterer Einzelheiten wird auf die Erstpublikation verwiesen [8].

Nach diesen Kriterien kann ein Vergleich der Funktionsparameter mit Referenzwerten erfolgen. Der Gutachter kann die aktuelle Situation des von ihm begutachteten Probanden mit konkreten guten und schlechten Ausheilungsergebnissen in der von ihm zu beurteilenden Region vergleichen und dann schlüssig seine Invaliditätsbemessung begründen. Die Referenzwerte der Bemessungsempfehlung sind zu erreichen unter: www.invaliditaet-online.de.

Abschließend sei noch einmal darauf hingewiesen, dass keine Invaliditätswerte anhand einzelner Messparameter zu bestimmen sind, wie z. B. „ab einem ∆GDW von x oder y resultiert eine ‚Mindest‘-Invalidität von z. B. z“. Dafür sind die Einflussfaktoren auf die Funktionsbeeinträchtigungen des Achsorgans zu vielschichtig.

### Becken

Im Bereich des Beckens können nach Verletzungen regelhaft auch Unfallverletzungsfolgen, die einer gutachtlichen Untersuchung nur schwer zugänglich sind, bestehen bleiben. Bei gesicherter Erstgesundheitsschädigung sind einerseits die bildgebenden Veränderungen wie z. B. Knochennarben oder magnetresonanztomo-/szintigraphische Nachweise von Reizzuständen zu beschreiben, und andererseits ist der ärztliche Sachverständige gehalten, die in diesem Zusammenhang nachweisbaren Funktionsbeeinträchtigungen möglichst genau zu beschreiben und einer Plausibilitätsprüfung zu unterziehen. Der Gutachter muss also subjektive Beschwerdeangaben an nachweisbaren Funktionsbeeinträchtigungen plausibel machen oder bei fehlendem Nachweis von funktionellen Beeinträchtigungen ausschließen:

- verkürzte Dauer des erträglichen Sitzens,
- Sitzimbalance mit Angewiesensein auf ein orthopädisches Sitzkissen oder einen orthopädischen Bürostuhl,
- Verminderung der Gehstrecke,
- Auftreten einer belastungsabhängigen Gangbildstörung durch zunehmende Schmerzen im Becken,
- ausstrahlende Schmerzen in die Lendenwirbelsäule und in die Hüften durch eine fehlende Beckenrotation (Versteifung der Kreuz-Darmbeingelenk-Fugen),
- Unmöglichkeit des Einnehmens von Zwangshaltungen mit tiefem Hocksitz oder weitem Vornüberbeugen durch dabei auftretende Zugspannungen im aus der Verletzung resultierenden knöchernen und Weichteilnarbengewebe,
- Beinlängenunterschied durch Fehlstellung.

Diese Funktionsbeeinträchtigungen müssen durch möglichst viele bildgebende *und* klinische Befunde gestützt oder widerlegt werden:

- Ausbildung der Gesäßmuskulatur,
- Weichteilgrübchen,
- Asymmetrie der Beinmuskulatur,
- Fußsohlenbeschwielung,
- Verknöcherung der ISG-Fugen bzw. Osteophytenbildungen,
- radiologisch nachweisbare Beckenasymmetrie (auch dynamisch durch Aufnahmen bei wechselndem Einbeinstand),
- im MRT Zeichen des chronischen Reizzustands i. B. der Schoßfuge oder der Kreuzbein-Darmbein-Gelenke,
- bildgebende Veränderungen am lumbosakralen Übergang,
- Schädigung des N. pudendus (Schamnerv),
- Piriformis-Syndrom (Schmerzen und Taubheitsgefühle aufgrund einer Einengung des Ischiasnervs durch den Piriformis-Muskel).

Auch im Bereich des Beckens gilt, dass bei normüberschreitenden nozizeptischen und/oder neuropathischen Schmerzen eine neurologische und/oder psychiatrische Zusatzbegutachtung veranlasst werden sollte (Tab. 19).

**Tab. 19:** Beurteilung von Unfallfolgen am Becken

Unfallfolgen	Invalidität je nach Ausprägung der Funktionsbeeinträchtigung (%)
Beckenasymmetrie (umfassende radiologische Diagnostik notwendig)	10–20
Verknöcherung oder Reizzustand der Schoßfuge oder der Kreuzdarmbeingelenke	0 bis ≤ 10
Symphysale Diastase > 15 mm	5–10
Atrophes/instabiles Falschgelenk im Bereich des Scham- oder Sitzbeins	0–5
Atrophes/instabiles Falschgelenk, vorderer Beckenring einseitig	Um 15
Atrophes/instabiles Falschgelenk, vorderer Beckenring beidseitig	Um 20
Unfallfolgen wie ein Piriformis-Syndrom oder eine Schädigung des N. pudendus sind Einzelfälle, sie sind zusammen mit neurologischen und ggf. urologischen Zusatzgutachter zu beurteilen	

### Brustkorb, Brustbein, Rippen

Bei stabil verheilten Brüchen des Brustbeins ohne erkennbare Knochennarbe resultiert regelhaft keine messbare Invalidität. Bei Ausheilung mit Achsenknick je nach Ausprägung der Funktionsbeeinträchtigung ist eine Invalidität um 5 % zu erwarten.

Knöchern ohne jegliche erkennbare Knochennarben oder Fehlstellung ausgeheilte Rippenbrüche lassen regelhaft eine messbare Invalidität nicht begründen. Ist aber röntgenmorphologisch eine funktionell relevante Fehlstellung oder ein Falschgelenk vorhanden oder die Irritation der interkostalen Nerven nachzuweisen, so ist je nach Ausdehnung (eine bis 2 Rippen oder Rippenserienbruch) die Invalidität bis ≤ 10 % zu begründen. Fehl- oder falschgelenkig verheilte Rippenbrüche nach Rippenserienbruch mit erkennbarer Deformierung des Brustkorbes sind bei nachgewiesener Störung der Atemmechanik mit 10 % zu bemessen bei interpolierender Betrachtung der Lungenfunktionsstörung. Es ist also in diesen Fällen (und das auch insbesondere bei Schwielen- und Schwartenbildungen) eine fachinternistische Lungenfunktionsdiagnostik/Zusatzbegutachtung erforderlich. Diesbezüglich sind auch Folgen von Blut- oder Luftansammlungen zwischen Lunge und Brustkorbwand regelhaft fachinternistisch mitzubeurteilen (Tab. 20).

**Tab. 20:** Bemessung von Unfallfolgen am Brustkorb, -bein und den Rippen

Funktionsbeeinträchtigungen im Bereich Brustkorb/Brustbein/Rippen	Invaliditätseckwert (%)
Mit Achsenknick verheilter Brustbeinbruch je nach Funktionsbeeinträchtigung	Um 5
In Fehlstellung oder falschgelenkig verheilter Rippenbruch je nach Funktionsbeeinträchtigung (eine bis 2 Rippen oder Rippenserienbruch)	0 bis ≤ 10
Fehl- oder falschgelenkig verheilte Rippenbrüche nach Serienbruch mit erkennbarer Deformierung des Brustkorbes bei nachgewiesener Störung der Atemmechanik	10

### Bauchdecke

Reizlos und stabil verheilte Bauchwandnarben nach Bauchöffnung führen regelhaft nicht zu funktionellen Beeinträchtigungen.

Liegen narbige Umbildungen im Sinne eines Keloids oder auch Verwachsungsbeschwerden vor, so sind bei nachgewiesenen Funktionsbeeinträchtigungen (z. B. mit fotografischen und/oder sonographischen/magnetresonanztomographischen Befunden) Invaliditätswerte bis 5 % zu rechtfertigen.

Bei großen Bauchwandhernien kommt es bereits bei der normalen Bauchpresse zum Austritt von Eingeweiden, sodass regelhaft das Tragen eines Bruchbandes bereits bei normalen Verrichtungen des täglichen Lebens erforderlich ist. Allerdings spielen in der PUV Hilfsmittel mit Ausnahme der Brille/Kontaktlinse keine Rolle, deshalb ist die Größe/Ausdehnung des Bauchwandbruchs invaliditätsrelevant. Andererseits führen Vorwölbungen von Eingeweiden bei kleineren Bauchwandhernien eher zu einer Einklemmung. Ein solcher Zustand ist aber regelhaft eine Operationsindikation und wird dementsprechend nicht als Unfallverletzungsfolgezustand zu bewerten sein. Insofern bleibt unter funktionellen Gesichtspunkten lediglich die „funktionelle“ Abstufung anhand der Größe des Bauchwandbruchs (Tab. 21).

**Tab. 21:** Bemessung von Unfallfolgen im Bereich der Bauchdecke

Funktionsbeeinträchtigungen im Bereich Bauchdecke	Invalidität (%)
Narbige Umwandlungen eines Teiles der Bauchwandmuskulatur	≤ 5
Reponible Bauchwandhernie bis Tischtennisballgröße	≤ 10
Reponible Bauchwandhernie bis Faustgröße	≤ 15
Reponible Bauchwandhernie über Faustgröße	≤ 20

### Verbrennungs‑/Verbrühungs‑/Verätzungsfolgen/-narben

Zu diesem Komplex können keine allgemein gültigen Eckwerte einer Invaliditätsbemessung angegeben werden, da die Folgen von Verbrennungen und/oder Verbrühungen weit gestreut sind. Es müssen aber Kenntnisse aus den ärztlichen Behandlungsdokumentationen über den Schweregrad und die Ausdehnung der Primärverletzung vorliegen. Erst dann ist es auch möglich, die Haut als Organ des Körpers zu begreifen mit ihrer sowohl äußeren Schutzfunktion als auch ihrer Mitbeteiligung an der Regulation des Temperatur‑, Flüssigkeits- und Elektrolythaushalts [10]. Kann sich also der Betroffene noch Temperaturschwankungen aussetzen oder ist ihm das aufgrund der Temperaturregulationsstörung verwehrt? Auch müssen ggf. vorliegende taktile Funktionsstörungen, Störungen über die der originären Hautfunktion hinausgehende Folgen z. B. durch Narbenstränge mit Störungen der Gelenkbeweglichkeit Berücksichtigung finden.

Zur Beschreibung der Narben müssen Aussagen getroffen werden über

- die Pigmentierung des Narbenareals,
- die Höhe der Narbe über Hautniveau,
- ihre Textur, Stabilität und Dehnbarkeit.

Weiter sind Informationen notwendig über

- die Durchblutung,
- die Plausibilität eines Juckreizes oder der
- Störung der Schweißsekretion.

All diese und ggf. noch weitere Parameter müssen beschrieben und diskutiert werden, bevor man das Gesamtbild dann mit Funktionsbeeinträchtigungen inner- und außerhalb der Gliedertaxe vergleicht. Dies macht es dem Gutachter möglich, eine plausible und transparente Invaliditätsbemessung vorzunehmen.

Punktesysteme, wie sie zur Schätzung der MdE vorgeschlagen werden [10], gaukeln nur eine mathematische Genauigkeit vor und können bei der Invaliditätsbemessung keine Anwendung finden.

#### Addendum

Nach der BGH-Rechtsprechung vom 01.04.2015 soll die Schulter nicht zum Arm gehören. Danach müsste die Invalidität, wie in Tab. 22 dargestellt, außerhalb der Gliedertaxe bemessen werden.

**Tab. 22:** Bemessung von Schulterfunktionsbeeinträchtigungen außerhalb der Gliedertaxe, inkl. Versteifung

Schulterfunktionsbeeinträchtigungen außerhalb der Gliedertaxe, inkl. Versteifung	(%)
Schultergelenkversteifung	3
Einschränkung der Vorhebung bis 120°	1
Einschränkung der Vorhebung bis 90°	2
Einschränkung der Vorhebung bis 60°	2
Einschränkung der Vorhebung bis 30°	3
Persistierende Schultereckgelenkinstabilität Rockwood 2 oder höher je nach individuellem Funktionsdefizit im Vergleich zu anderen Eckwerten von Schulterfunktionsbeeinträchtigungen	2
Verformung/Subluxation im Schlüsselbein‑/Brustbeingelenk mit klinischer Symptomatik	3

## Fazit für die Praxis

- Diese Bemessungsempfehlungen lösen die bisher allgemein Anerkennung findenden Eckwerte von Schröter und Ludolph aus 2009 ab.
- Die Invaliditätseckwerte sind (*beachte*: erstmals) fach- und länderübergreifend konsentiert.
- Online (invaliditaet-online.de) erfolgen regelmäßig eine Evaluierung und ggf. Anpassung, sodass der Anwender immer den neuesten Stand einsehen kann.
- Die Online-Visualisierung unter invaliditaet-online.de erleichtert das Verständnis für die Bewertung von Funktionsstörungen auch für Nichtmediziner wie Richter, Rechtsanwälte und Sachbearbeiter.

## Abkürzungen

A: Armwert
AUB: Allgemeine Unfallversicherungsbedingungen
B: Beinwert
BGH: Bundesgerichtshof
BW: Brustwirbel
D: Daumenwert
DGUV: Deutsche Gesetzliche Unfallversicherung
F: Fußwert
FGIMB: Fachgesellschaft Interdisziplinäre Medizinische Begutachtung e.V.
Fi: Fingerwert
GUV: Gesetzliche Unfallversicherung
Gz: Großzehenwert
H: Handwert
HW: Halswirbel
HWK: Halswirbelkörper
LW: Lendenwirbel
MdE: Minderung der Erwerbsfähigkeit
PUV: Private Unfallversicherung
SW: Sakralwirbel
UA: Unterarm
Z: Zehenwert
∆GDW: Delta des Grund-Deckplatten-Winkels
